# Habitat selection and seasonal movements of young bearded seals (*Erignathus barbatus*) in the Bering Sea

**DOI:** 10.1371/journal.pone.0192743

**Published:** 2018-02-28

**Authors:** Michael F. Cameron, Kathryn J. Frost, Jay M. Ver Hoef, Greg A. Breed, Alex V. Whiting, John Goodwin, Peter L. Boveng

**Affiliations:** 1 Marine Mammal Laboratory, Alaska Fisheries Science Center, NOAA, National Marine Fisheries Service, Seattle, Washington, United States of America; 2 School of Fisheries and Ocean Sciences, University of Alaska-Fairbanks, Fairbanks, Alaska, United States of America; 3 Institute of Arctic Biology, University of Alaska-Fairbanks, Fairbanks, Alaska, United States of America; 4 Native Village of Kotzebue, Kotzebue, Alaska, United States of America; Sanya Institute of Deep-sea Science and Engineering Chinese Academy of Sciences, CHINA

## Abstract

The first year of life is typically the most critical to a pinniped’s survival, especially for Arctic phocids which are weaned at only a few weeks of age and left to locate and capture prey on their own. Their seasonal movements and habitat selection are therefore important factors in their survival. During a cooperative effort between scientists and subsistence hunters in October 2004, 2005, and 2006, 13 female and 13 male young (i.e., age <2) bearded seals (*Erignathus barbatus*) were tagged with satellite-linked dive recorders (SDRs) in Kotzebue Sound, Alaska. Shortly after being released, most seals moved south with the advancing sea-ice through the Bering Strait and into the Bering Sea where they spent the winter and early spring. The SDRs of 17 (8 female and 9 male) seals provided frequent high-quality positions in the Bering Sea; their data were used in our analysis. To investigate habitat selection, we simulated 20 tracks per seal by randomly selecting from the pooled distributions of the absolute bearings and swim speeds of the tagged seals. For each point in the observed and simulated tracks, we obtained the depth, sea-ice concentration, and the distances to sea-ice, open water, the shelf break and coastline. Using logistic regression with a stepwise model selection procedure, we compared the simulated tracks to those of the tagged seals and obtained a model for describing habitat selection. The regression coefficients indicated that the bearded seals in our study selected locations near the ice edge. In contrast, aerial surveys of the bearded seal population, predominantly composed of adults, indicated higher abundances in areas farther north and in heavier pack ice. We hypothesize that this discrepancy is the result of behavioral differences related to age. Ice concentration was also shown to be a statistically significant variable in our model. All else being equal, areas of higher ice concentration are selected for up to about 80%. The effects of sex and bathymetry were not statistically significant. The close association of young bearded seals to the ice edge in the Bering Sea is important given the likely effects of climate warming on the extent of sea-ice and subsequent changes in ice edge habitat.

## Introduction

Bearded seals (*Erignathus barbatus*) are a key ecological component of Arctic and sub-Arctic marine ecosystems, yet few details of their ecology have been documented in the scientific literature. Subsistence hunters residing in indigenous coastal communities have, for millennia, harvested this large seal for nutritional and cultural needs. While the traditional knowledge about bearded seals accumulated through generations of reliance on this resource is quite detailed for times and areas where seals are harvested, it is incomplete for other times of year and areas far offshore. Compared to most other pinnipeds, bearded seals are particularly difficult to approach, capture and handle for scientific purposes, especially in locations where they are regularly hunted. Recent collaborative efforts between researchers and Alaska Natives, however, have led to the development of effective capture and handling techniques for young bearded seals. As a result, the opportunities for ecological study, and the employment of advanced monitoring technologies such as satellite telemetry, have been greatly improved [1–3]. Bearded seals inhabit shallow, seasonally ice-covered waters. These seals generally occupy ice habitat that is broken and drifting, with natural areas of open water such as leads, fractures, and polynyas, which the seals use for breathing and for access to water for foraging [4–7]. Sea-ice is important to bearded seals throughout the year as a platform for resting and perhaps thermoregulation [8]. It likely also provides some protection from marine predators [9], and allows molting seals a dry location to raise their skin temperature and facilitate epidermal growth [10]. Bearded seals usually avoid areas of shorefast ice that is thick and continuous and they are also rarely seen in the vicinity of drifting ice that is heavy and unbroken or in large areas of multi-year ice [5, 6, 11–15]. Although they are known to include schooling pelagic fishes in their diet when advantageous [16, 17], bearded seals feed primarily on benthic organisms that are more numerous in shallow water where light can reach the seafloor [12, 18]. As such, their effective range is typically restricted to areas where seasonal sea-ice occurs over relatively shallow waters [4–7, 11, 19–21].

The shallow shelf of the Bering and Chukchi seas provide the largest continuous area of favorable foraging habitat for bearded seals [5, 11, 22]. These continental shelves are typically covered by sea-ice in late winter and spring and are mostly ice free in late summer and fall, a pattern that is believed to drive seasonal movements and distribution of bearded seals in this area [7, 11, 22]. During winter, the favorable conditions of shallow waters combined with broken, drifting and fractured pack ice occur more often in the Bering Sea than the Chukchi Sea [11]. These conditions may be the reason that the central and northern parts of the Bering Sea shelf have the highest densities of bearded seals in winter [4, 5, 11, 23–25]. As the ice retreats in the spring, most adults in the Bering Sea are thought to move north through the Bering Strait. There they spend the summer and early fall at the southern edge of the Chukchi and Beaufort Sea pack ice and at the wide, fragmented margin of multi-year ice [4, 5, 7, 11, 23, 25]. A smaller number of bearded seals, mostly juveniles, remain near the coasts of the Bering and Chukchi seas during summer and early fall instead of moving with the ice edge and are often found in bays, estuaries and river mouths [4, 11, 22]. As the ice forms again in the fall and winter, most bearded seals are thought to move south again with the advancing ice through Bering Strait returning to the Bering Sea where they spend the winter [7].

For pinnipeds, the first year of life is typically the most critical to long-term survival. This is especially true for Arctic phocids like the bearded seal that are abruptly weaned at only a few weeks of age and must learn to locate and capture prey on their own before blubber stores accumulated during nursing are depleted [1–3]. About 40% of bearded seal pups survive to age 1, and only 28% live to age 3 [7]. Proficiency at feeding, and therefore habitat selection and seasonal movements, are important to their survival, and a better understanding of these factors is critical for developing sound conservation and management plans.

**Fig 1:**
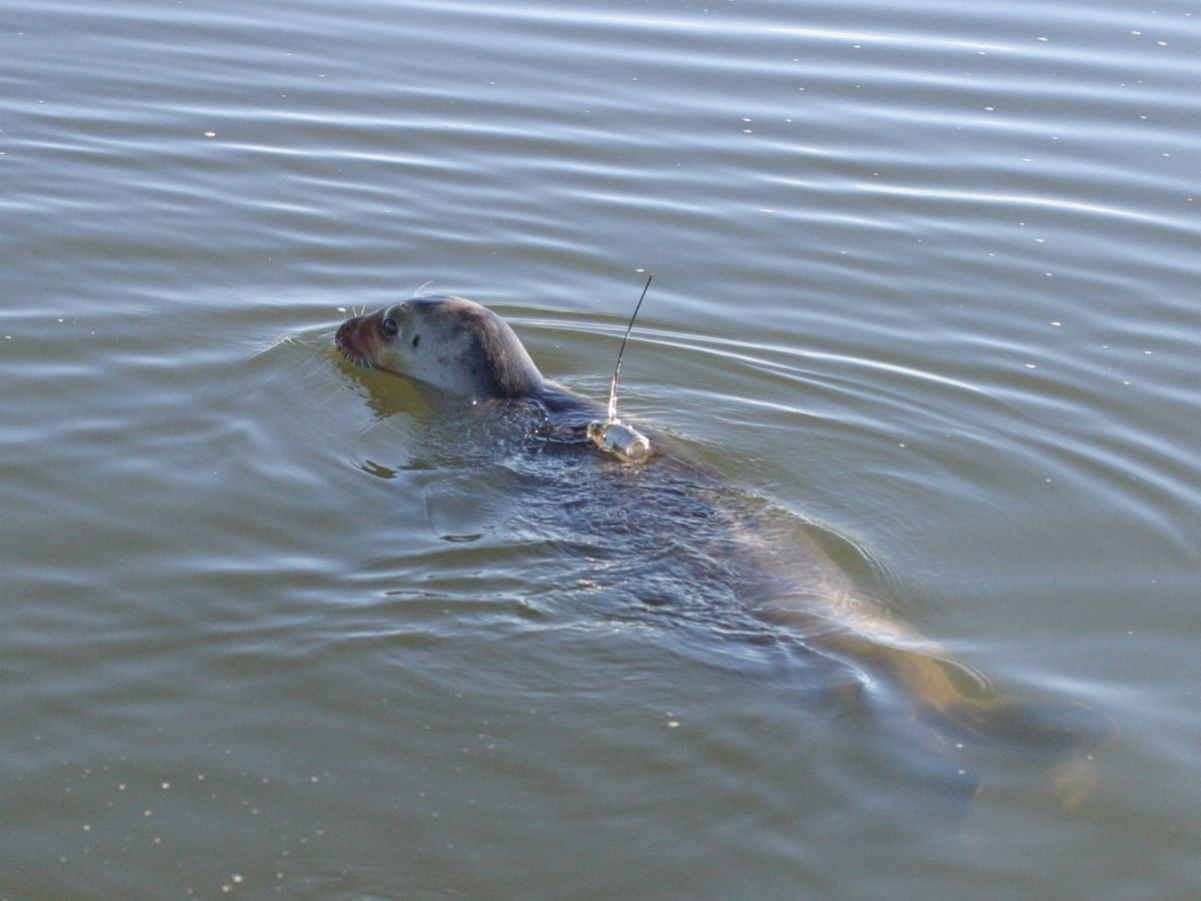
Young-of-year bearded seal released into Kotzebue Sound, AK, with a SPLASH satellite-linked dive recorder (SDR) attached to its back.

This study was motivated by the importance of bearded seals to the Arctic marine ecosystem, including its human inhabitants; potential impacts of oil and gas development and other human activities on bearded seal habitat; concerns about bearded seals’ habitat in a disrupted, warming climate; and the need for better information to support decisions about this protected species. These are some of the same concerns that led to a review of the bearded seals’ conservation status [26] and listing of the species as “threatened” in U.S. waters, under the Endangered Species Act (ESA) [27]. In this paper, our goals are to describe the seasonal movements of young bearded seals in Alaska as determined from satellite-linked dive recorders (Fig 1) and to identify important components of their habitat selection.

## Materials and methods

We used satellite telemetry to record locations of young bearded seals in the Bering Sea. We analyzed the seals’ locations with a movement model, used the results to simulate animal locations from a ‘null’ movement model, and then used logistic regression as a resource selection function (RSF) [28] to compare habitat variables for observed animal locations to those for simulated locations. All statistical analyses were performed using R [29]. Below we provide specific details on each step of our analysis.

### Data collection and study region

**Fig 2:**
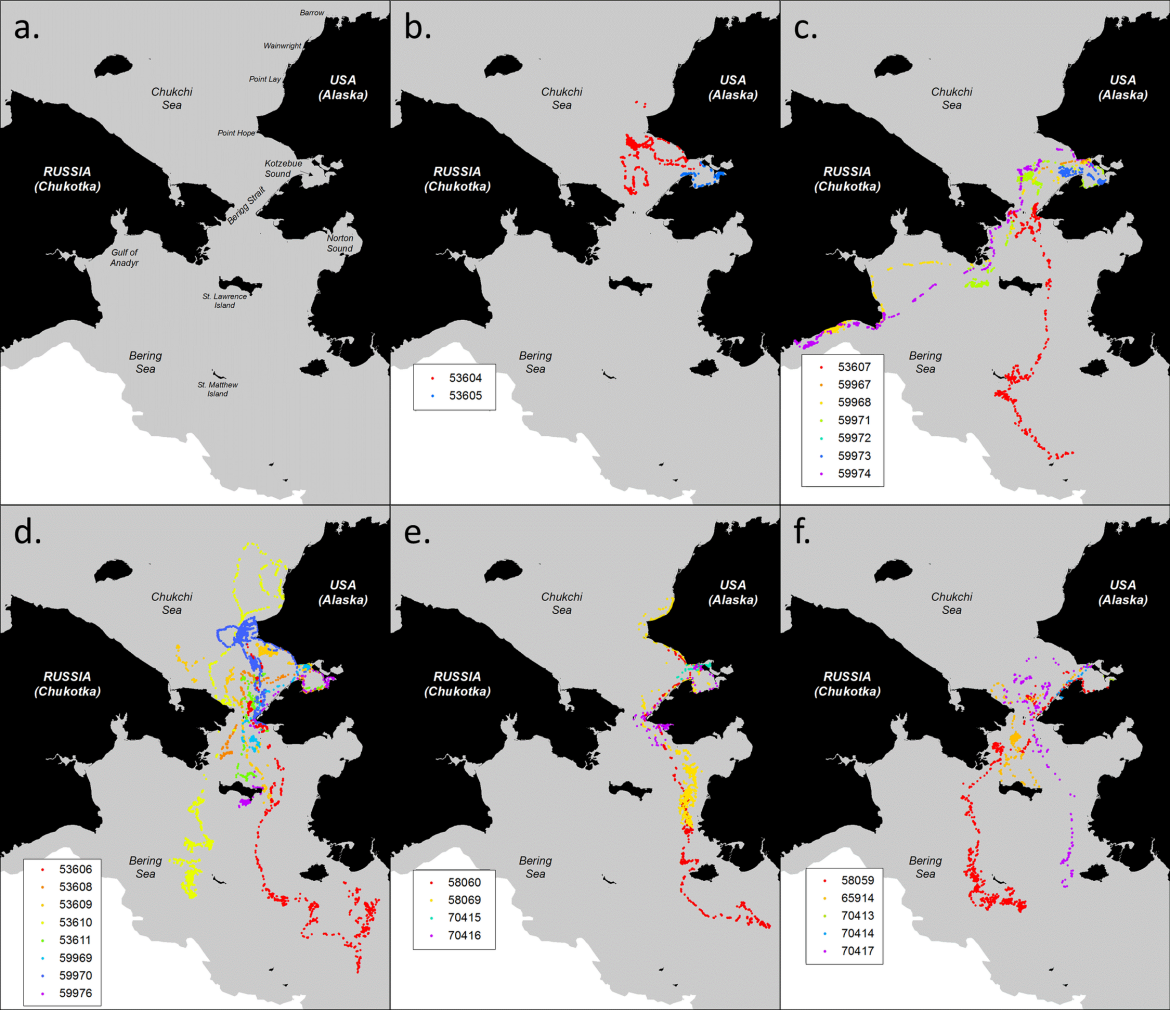
Maps a) of the Chukchi and Bering Sea Shelf region; and of the reported locations of bearded seals tagged in Kotzebue Sound in autumn: b) 2004-females, c) 2005-females d) 2005-males, e) 2006-females, f) 2006-males. The gray polygon indicates water depths < 1000 m.

Kotzebue Sound (Fig 2A), an embayment of the southeastern Chukchi Sea, was selected as the tagging location for this study. The Native Village of Kotzebue is one of many communities along the coast of Alaska where bearded seals remain important for subsistence purposes. The hunters there are aware that young-of-the-year bearded seals occupy areas near that coast well into the fall [30], and the community has a history of supporting and participating in research on species important to the region.

During September-October of 2004–2006, researchers and Alaska Native hunters captured, instrumented and released 26 (13 female and 13 male) young bearded seals with satellite-linked dive recorders (SDRs) in Kotzebue Sound, Alaska (Table 1). There is considerable uncertainty associated with the field classification of age in young bearded seals. Yet, based on their standard lengths [31] and the frequent presence of a characteristic “T” shaped hair coloration on their forehead, all captured seals were thought to be less than 2 years old, with most being young-of-the-year animals (i.e., approximately 6 months old). Seals were captured using specially designed large-mesh (12 inch stretched) nylon twine nets. A net was composed of 1 to 3 panels, each 90 ft. long and 12 ft. or 24 ft. deep. The float line was made of a ¾ inch dia. foam core wrapped in nylon and the leaded line at the bottom of the net was ¼ inch diameter; this lightweight lead line ensured that entangled seals could reach the surface to breathe. The nets were set perpendicular to the coastline in shallow, ice-free waters where young bearded seals were known to travel and feed prior to freeze-up.

**Table 1:** Information on the 26 seals (and their SDRs) in this study.

SDR-ID	SDR-type	Sex	Standard length (cm)	Axillary / maximum girth (cm)	Date captured	Date entered Bering Sea*	Date of last SDR location	Duration (days) of SDR record	Days with SDR location	Location days in Bering Sea*	Used in habitat analysis
53604	SPLASH	F			5-Oct-04	n/a	1-Feb-05	120	90	0	
53605	SPLASH	F			11-Oct-04	n/a	2-Dec-04	53	49	0	
53606	SPLASH	M	148	113 / 123	24-Sep-05	10-Nov-05	1-Apr-06	190	157	141	X
53607	SPLASH	F	141	97 / 110	24-Sep-05	16-Nov-05	8-Feb-06	138	105	84	X
53609	SPLASH	M	140	123 / 122	26-Sep-05	n/a	5-Jun-06	253	149	0	
53608	SPLASH	M	142		30-Sep-05	9-Nov-05	26-Nov-05	58	55	18	X
53610	SPLASH	M	166		1-Oct-05	21-Dec-05	30-Apr-06	212	176	109	X
53611	SPLASH	M	141		2-Oct-05	2-Dec-05	15-Jan-06	106	84	33	X
59971	SPLASH	F	143	115 /	2-Oct-05	2-Dec-05	27-Dec-05	87	81	24	X
59970	SPLASH	M	129		3-Oct-05	n/a	20-Apr-06	200	170	0	
59972	SPLASH	F	149	110 / 115	4-Oct-05	9-Nov-05	30-Mar-06	178	176	141	X
59968	SPLASH	F	135		7-Oct-05	27-Oct-05	4-May-06	210	168	148	X
59969	SPLASH	M	145	121 / 125	7-Oct-05	22-Oct-05	19-Nov-05	44	39	25	X
59973	SPLASH	F	134		7-Oct-05	n/a	7-Jan-06	93	77	0	
59974	SPLASH	F	140	96 / 106	7-Oct-05	7-Nov-05	22-Feb-06	139	95	67	X
59976	SPLASH	M	157	129 / 131	7-Oct-05	20-Oct-05	2-Feb-06	119	82	66	X
59967	SPLASH	F	126		11-Oct-05	n/a	15-Oct-05	5	5	0	
58069	SPLASH	F	150	113 / 124	3-Oct-06	21-Nov-06	26-May-07	236	216	172	X
70413	CTD	M	138	102 / 118	6-Oct-06	n/a	9-Oct-06	4	2	0	
58059	SPLASH	M	134	102 / 114	7-Oct-06	11-Nov-06	4-May-07	210	190	162	X
70414	CTD	M	136	108 / 114	7-Oct-06	n/a	12-Oct-06	5	5	0	
70415	CTD	F	139	108 / 120	7-Oct-06	n/a	26-Oct-06	19	14	0	
70416	CTD	F	144	118 / 138	7-Oct-06	8-Nov-06	18-Dec-06	73	57	28	X
70417	CTD	M	151	120 / 133	7-Oct-06	7-Dec-06	31-Dec-06	86	62	22	X
58060	SPLASH	F	137	116 / 128	20-Oct-06	12-Nov-06	18-Jan-07	91	88	68	X
65914	SPLASH	M	148	100 / 109	22-Oct-06	6-Nov-06	22-Dec-06	62	60	46	X

* We defined the northern boundary of the Bering Sea as latitude N 65° 45’. Some seals crossed this boundary more than once. As such, the date of entering the Bering Sea was defined as the last date a seal crossed south of N 65° 45’ before its SDR ceased transmitting (e.g., the battery failed or the seal molted).

Entangled seals were removed from the net and placed in hoop nets on the deck of a boat or on the beach until they were processed. Captured seals were measured, and samples of their blood (< 40 ml), skin and blubber (approximately 500 mg total), were collected to establish baseline parameters for health and condition and for DNA studies. Seals were instrumented with one of two SDRs: a SPLASH tag (manufactured by Wildlife Computers, Redmond, WA, USA) or a CTD tag (manufactured by SMRU, St. Andrews, Fife, Scotland). The SDRs were attached to the hair on the seals’ backs using Devcon quick-setting epoxy (Fig 1) and were expected to fall off during the seals’ annual molt the following spring, after providing information for up to 8 months. The SDRs collected information on the timing and depth of seals’ dives and their haul-out timing. Locations of the tagged seals were determined by the Argos Data Collection and Location System (http://www.argos-system.org/ operated by CLS [32]), which also relayed dive and behavior data that were recorded by the tags. These data were processed through Wildlife Computers’ DAP software or by SMRU and all data were uploaded to an Oracle database for additional processing and long-term archival.

Locations provided by the Argos System are calculated using Doppler shift measurements from multiple SDR transmissions received during a single satellite overpass. The accuracy of the location is classified with a Location Quality (LQ) of 3, 2, 1, 0, A, B or Z, with 3 expected to have the smallest error, B the greatest, and Z considered an “invalid location” due to insufficient uplinks [33]. Although more sophisticated methods have since been developed for identifying unlikely ARGOS locations based on movement rates and turning angles [34, 35], we simply filtered out the worst quality locations (i.e., LQ = B and Z), and removed those few remaining locations that placed the seal on land (as defined by the AMSR-E grid of the Bering Sea used to define our study area). We plotted (Fig 2) all remaining locations using a Geographic Information System (ArcGIS Desktop, produced by ESRI, Redlands, CA, USA) to visually identify and compare gross movement patterns among seals.

After being released, most seals remained in the ice-free waters of the Chukchi Sea for only 1 to 2 months before heading south through the Bering Strait with the advancing sea-ice. For completeness and context we describe these Chukchi Sea movements in the results presented below. Data from the Chukchi Sea were insufficient for RSF analysis however (few data points over very narrow temporal window), so we restricted our RSF selection analysis to include only those 17 seals (8 female and 9 male) that entered the Bering Sea (defined as south of 65°45’N) and remained there until their SDRs ceased transmitting (i.e., the battery failed or the seal tag was shed (Table 1)).

### Analysis and simulation of bearded seal movements

We used logistic regression to fit a RSF in a matched case-control approach [36]. For our study, the observed seal locations were the “case,” and we obtained a matched sample from available habitat as the “control”. Spatio-temporal autocorrelation tends to bias downward the variances of estimated RSF parameters however, which in turn biases the analysis to infer too many spurious relationships [37, 38]. Hence, we modified the use of logistic regression as an RSF [28] by adding a Monte Carlo randomization to obtain correct variances [39]. In this section, we describe the Monte Carlo randomization, which first required an analysis of movements (step lengths and turning angles) of tagged seals using a generalized linear model.

Models of, and methods to analyze, animal movement are advancing rapidly [40–46]. Some of these new approaches attempt (with varied levels of success) to incorporate habitat into fitted movement models (e.g. [43, 47, 48]) or to relate movement to habitat [49]. However, our goal was to simulate movement in the absence of environmental covariates that might otherwise influence movement so that this simulated movement could be compared to real tracks as matched case-controls for a RSF. Many of the models listed above are elegant and allow predictions in space at unsampled times. However, they are computationally demanding and are very difficult to use when analyzing many animals simultaneously. Our analytical approach is statistically sound and pragmatic for our data set.

Animal tracks can be represented as sequential movement vectors, each characterized by elapsed time, bearing, and length from which secondary components can be calculated (e.g., dividing length by elapsed-time yields average speed). Before simulating movements, we fit several generalized linear models (GLMs) to estimate transition probability tables for directional bearings and distances moved from real observations (see S1 File for details). A correlated random walk (CRW) model was developed using data from seals (case) that were moving south to follow advancing sea ice during freeze-up. As such, there was also a southerly tendency in movement in the simulated locations (control). This tendency mimicked southerly movement of real seals such that controls remained a good proxy for available habitat as the winter season progressed. The southerly tendency in the CRW model from which simulations were produced, along with the constriction of the Bering Strait, effectively kept case-control locations in the Bering Sea.

The estimated movement parameters were used to simulate movements, creating locations from a null model (control) that matched the time increments from location to location and the sample size for each tagged seal, but with no habitat selection. Thus each real location for each tracked seal had an exactly temporally matched case-control. This temporal matching is somewhat less straightforward than one would intuitively expect because Argos error adds measurement error to the distribution of swimming speeds and turning angles that we needed for simulation. This contamination is increasingly serious as the simulation interval decreases, which we effectively addressed via a series of transformations to control skewed variance in the step length distribution attributable to this contamination (see S1 File for details).

**Fig 3:**
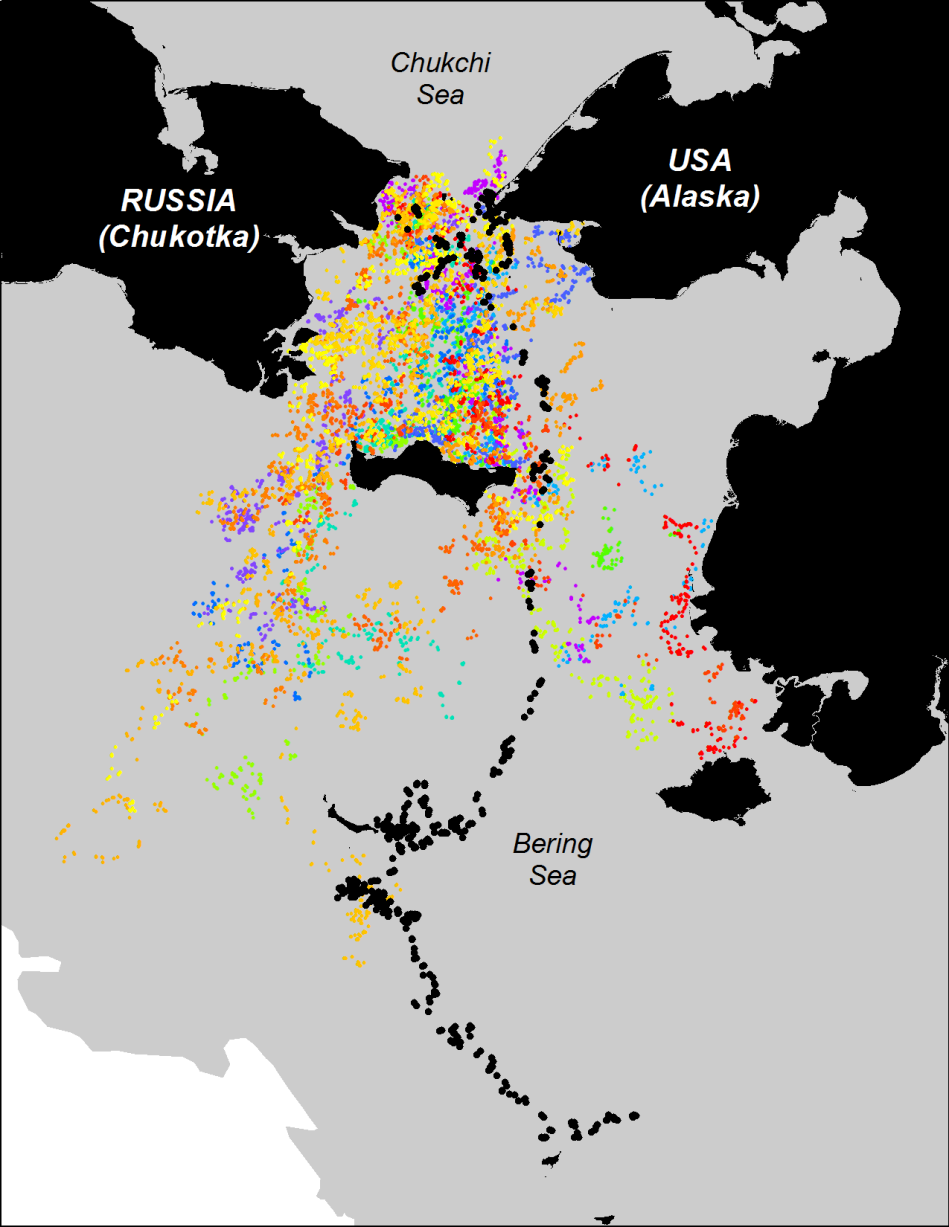
The movement track of seal 53607 in black, overlaid on the 20 simulated tracks paired for that individual (each simulation is a different color).

We simulated 20 independent case-control tracks for each of the 17 seals (see Fig 3; e.g., simulated tracks) which served as case-controls for 20 independent RSFs that form the nucleus of our Monte Carlo modification to the RSF. Although it would have been desirable to have more than 20 simulated tracks for the Monte Carlo estimates and tests [39], the computational time for each track was non-trivial. We used 20 tracks as the minimal number, based on the original suggestion by Barnard [50] and later Manly [39].

### Habitat covariates

We assigned habitat covariates to observed seal locations (case) and control locations from remotely sensed data. For each observed and simulated track, we obtained the covariates described in Table 2 at each location. We used the ETOPO2v2 Global Gridded database [51] to compute the variables related to seafloor depth. Depth was taken directly from the raster cell of an observed or simulated location. For d21kiso, we created an isobath of the 1000 m depths using the contour() function R [52] and then computed the shortest distance from each location to that isobath. We used the daily sea-ice raster images from the Advanced Microwave Scanning Radiometer—Earth Observing System (AMSR-E) [53] database to compute the variables related to sea ice. The variable ice_conc was taken directly from the raster cell of an observed or simulated location. We also included the quadratic form (i.e., ice_conc^2^) to allow for non-linear relationships. Similar to the 1000 m depth contour, we created a contour line of the 10% ice concentration to denote an ice edge. Although 10% concentration is a somewhat arbitrary threshold, it is appropriate to use a concentration > 0% to account for seasonal variability in measurement error at low ice concentrations [54]. Any contour of length less than 50 km was eliminated, and we computed the shortest distance from each location to the nearest of the remaining contours for the variable d2ice. Finally, we transformed the variables d2coast, d2ice, d21kiso and depth_m with their square roots to linearize their relationships to the response variable.

**Table 2:** The variables used in the model and their descriptions.

Variable	Description
d2coast	Distance (km) from the location to the nearest coastline
depth_m	Depth (m) of the seafloor at the location based on the ETOPO2v2 Global Gridded database
d21kiso	Distance (km) from the location to the 1000 m depth isobar, estimated from the ETOPO2v2 Global Gridded database
d2ice	Distance (km) from the location to ice edge (defined as the daily AMSR-E 10% ice concentration contour)
ice_conc	The daily AMSR-E percent ice concentration at the location
sea_ice	A binary variable (yes or no) indicating whether the location is in a field of sea-ice (based on the daily AMSR-E data) or in open water

### Resource selection via logistic regression

The locations of animal movements are correlated in both space and time. We estimated habitat selection parameters from the observed and simulated tracks using logistic regression in the form of a RSF, but we estimated standard errors via a Monte Carlo method. The logistic regression models were fit with maximum likelihood using the glm() function in R [29]. Note that we did not use a mixed model with a random effect for each seal. The simple logistic regression model weights each observation equally, allowing seals with more observations to have proportionally more weight. A mixed model would weight each animal more equally. While each approach has merits, we decided on the simple logistic regression. The glm() procedure that we used generally assumes independent observations, but our observations were not independent so the estimated standard errors would be incorrect due to autocorrelation. We obtained correct standard errors by Monte Carlo randomization, where we averaged the regression coefficients and used the standard deviations of the regression coefficients over the 20 Monte Carlo randomizations as our standard error terms. For the null hypothesis (i.e., regression coefficients are zero), we obtained the standard errors and *P*-values by assuming the mean regression coefficients were distributed normally with the standard deviation obtained from the 20 Monte Carlo fits of the logistic regression.

As traditional model selection techniques, such as AIC, are not available owing to the Monte Carlo modification of our RSF, we used a stepwise model selection procedure based on *P*-values to obtain a parsimonious model with all terms significant at *α* = 0.05. The initial model had all eight single effect covariates, an interaction between sea_ice and sqrt(d2ice), plus the interaction of sex with all other covariates and the sea_ice/sqrt(d2ice) interaction. We then removed interaction terms that were least significant one at a time. After removing insignificant interaction terms, we removed single effects that were least significant one at a time (unless they were part of a significant interaction term; see Table 3). The final model was interpreted as a resource selection function (e.g., [28], p. 103), where the regression coefficients indicated selection in the direction of the coefficient.

**Table 3:** These model factors and interactions were sequentially eliminated from the initial model at the given *P*-value.

Model factor	*P*-value
sex*ice_conc	0.3989
sex*sea_ice	0.3988
sex*sqrt(d2coast)	0.3964
sex*sqrt(d21kiso)	0.3911
sex*ice_conc^2^	0.3818
sqrt(d2coast)	0.3749
sex*depth_m	0.2960
sex*sea_ice*sqrt(d2ice)	0.3092
sex*sqrt(d2ice)	0.2252
depth_m	0.1354
sqrt(d21kiso)	0.1920
sex	0.0863

## Results and discussion

### Movements

Individual SDRs transmitted data for 4 to 253 days with a mean of 115 days (Table 1). Of the two female bearded seals tagged in 2004, one remained close to the coast of inner Kotzebue Sound and the other moved into the deeper waters of the Chukchi Sea (Fig 2B). Despite these different habitats, they both exhibited similar patterns in the use of their surroundings. Each seal tended to remain in a small area, presumably foraging, for a brief period (i.e., 3 to 18 days) before moving to a new location, often more than 150 km away. Neither of these seals entered the Bering Sea before their SDRs failed, and so they were not used in our habitat analysis.

In 2005, one female and four males (half of all males) headed north along the coast (Fig 2C and 2D). Each focused their movements for multiple days/weeks near Point Hope, an area known to be of high ocean productivity [55]. Two of these males continued farther north to Wainwright before turning around and heading south to the Bering Strait. Two females moved southeast into the shallow waters of inner Kotzebue Sound after being released (Fig 2C) and remained in shallow coastal waters until at least December when their SDRs failed. Most individuals traveled into the southern Chukchi Sea, and moved with the advancing ice into the Bering Sea. Four individuals (two males and two females) moved south of St. Lawrence Island (Fig 2C and 2D). The remaining two females moved west into Russian waters off the coast of the Gulf of Anadyr, towards the deeper waters of the northwest Bering Sea.

In contrast to 2005, young bearded seals tagged in 2006 did not focus their movements on any one location until they were south of Bering Strait (Fig 2E and 2F). All four females remained relatively close to the coast even after entering the Bering Sea, including one female that traveled as far north as Point Lay before returning southward. Although the males ranged widely, they tended to focus on the northern Bering Strait before entering the Bering Sea. Once in the Bering Sea, two males followed the advancing sea-ice south of St. Matthew Island, while the third remained in the heavier ice just north of St. Lawrence Island.

### Habitat selection analyses

The stepwise removal of effects, and the *P*-values for the removal, are presented in Table 3. The sex of a seal, and every interaction term that included sex, were not significant. This is an interesting result considering the observed differences in movement patterns and bearings between the sexes. Our analysis examines habitat however, not location or movement. Though it could be related to our small sample size, the lack of a significant sex-related effect in the final model suggests that males and females in this very young age class respond to habitat variables similarly, even if they move differently or are in different locations.

Depth is likely a critical habitat feature for bearded seals, which are predominantly benthic feeders. Unfortunately, none of our observed or simulated tracks extended off of the Bering Sea shelf for more than a few days, so we were unable to test for the effects of deeper water. That the variable ‘depth_m’ was not a significant term in our analysis supports the assumption that bearded seals are capable of exploiting the entire Bering Sea shelf [22].

**Fig 4:**
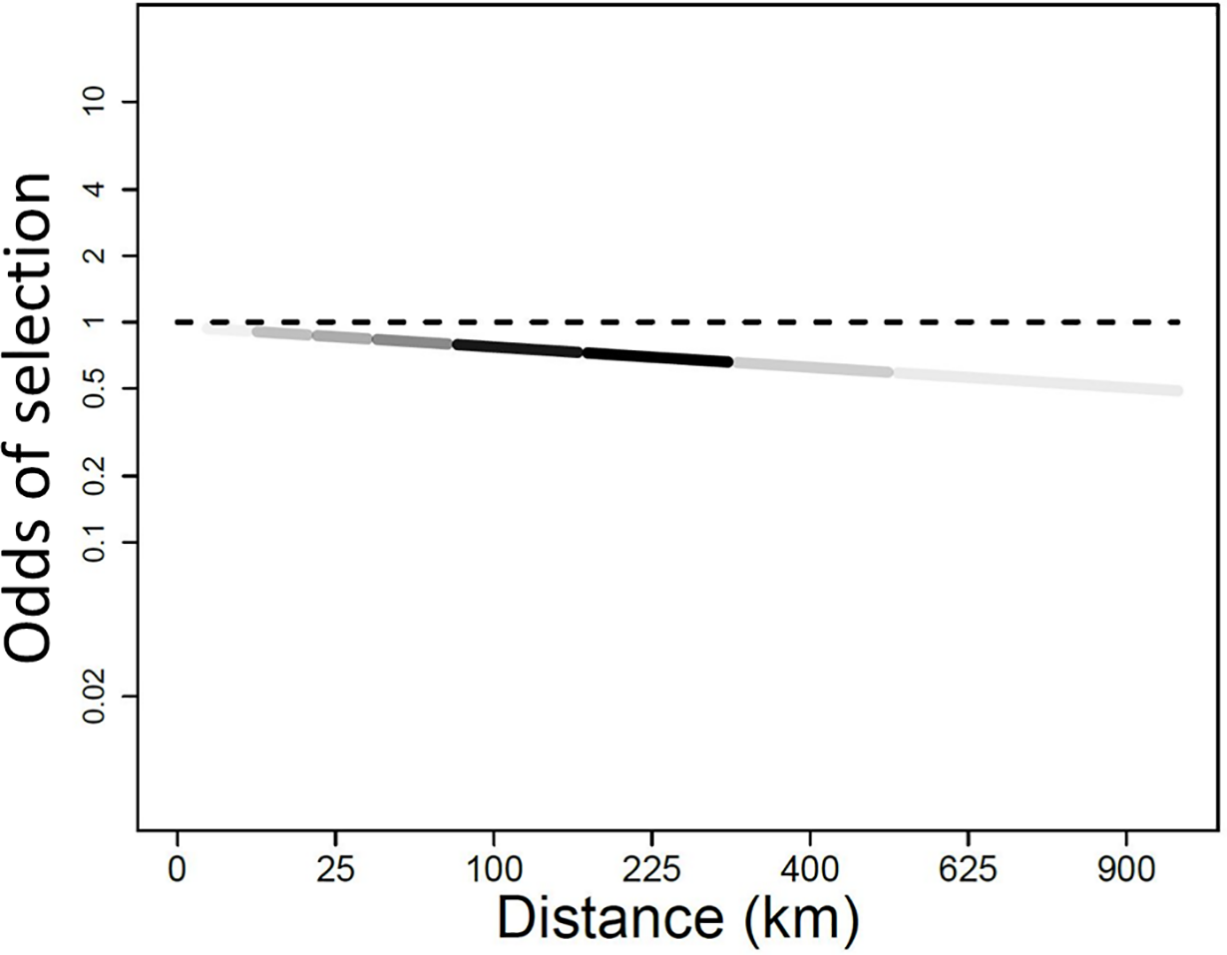
Graphical representation of model results for habitat locations in open water (i.e., sea_ice = no), as a function of distance from the ice. Values for odds of selection > 1 indicate chosen habitat and values < 1 indicate rejected habitat. The shading of the line represents the relative availability of the habitat to the tagged seal as sampled by the simulation. Young bearded seals did not select open water, but when in open water they chose locations closer to an ice edge (d2ice).

**Fig 5:**
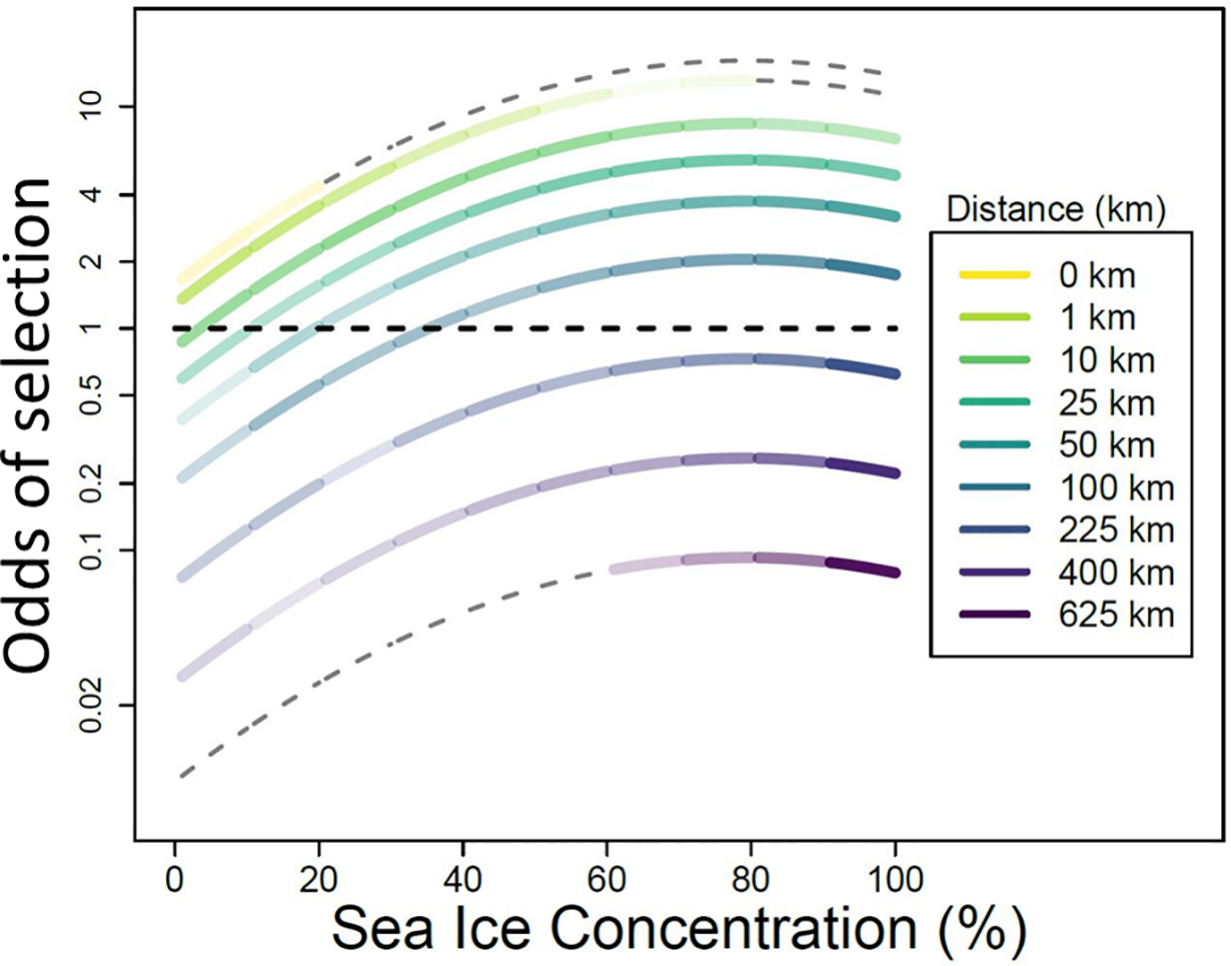
Graphical representation of model results for habitat within the sea-ice field (i.e., sea_ice = yes). Values for odds of selection > 1 indicate chosen habitat and values < 1 indicate rejected habitat. Individual curves represent the interactive effects of various distances to the ice edge (i.e., d2ice) and sea-ice concentration. The shading of each curve represents the relative availability of the given habitat to the tagged seal as sampled by the simulation. Gray dashes indicate no tagged seals occupied that habitat. In general, young bearded seals chose to be close to the edge and in higher ice concentrations, but the combination of these conditions are quite rare. For all distances, habitat selection peaked at about 80% ice concentration.

By modeling the log of the ratios of observed versus simulated locations, we effectively calculated the log odds of a tagged seal occupying a particular habitat versus a simulated seal, which had no influence of habitat characteristics on its movements. For graphical purposes we exponentiated model outputs to obtain the “odds of selection” [56]. An odds of selection > 1 indicates chosen habitat and an odds of selection < 1 indicates rejected habitat. The final model (Table 4) suggested that a key habitat selection factor was distance to an ice edge (i.e., sqrt(d2ice)); seals chose to be close to an ice edge. The strength of the relationship, however, depended on whether the seal was in open water (i.e., sea_ice = no) or within an ice field (i.e., sea_ice = yes). These young bearded seals did not select open water, but when in open water they chose locations closer to an ice edge (Fig 4). When in an ice field, however, their selection for ice edge habitat was much stronger, and the relationship fell off more rapidly with distance away from the edge (Fig 5).

**Table 4:** The terms and covariate estimates of the final habitat selection model. The estimates of logistic regression coefficients are on the logit scale and all terms are significant at α = 0.05.

Factor	Estimate	Std_Err	z_value	Prob z
Intercept	0.455066	0.235548	1.9320	0.0617
sea_ice = no	-0.501833	0.322640	-1.5554	0.1190
ice_conc	0.058692	0.010952	5.3592	< 0.0001
ice_conc^2^	-0.000370	0.000098	-3.7946	0.0003
sqrt(d2ice)	-0.206333	0.021916	-9.4149	< 0.0001
sqrt(d2ice)*(sea_ice = no)	0.185093	0.036084	5.1296	< 0.0001

The final model retained both the linear and quadratic forms of ice_conc (Table 4), and indicated that habitat selection of young bearded seals peaked at about 80% ice concentration. This may appear contradictory to a selection for ice edge habitat where concentration tends to be low. Our model results, however, must be interpreted in a multiple regression context; a model coefficient is the effect of the covariate with all other covariates in the model held constant. Ice concentration had a positive effect indicating that, on average, for any distance, seals tended to select this higher range of ice concentrations (Fig 5). Although our model predicts that young bearded seals would strongly select locations that are both close to the ice edge and in concentrations around 80%, the combination of these conditions are quite rare and nonexistent for simulated seal movements.

Our sea-ice habitat selection results are consistent with habitat associations found during springtime aerial surveys of the Bering Sea [57–59]. A small survey of the waters near St. Lawrence Island in March 2001 observed more bearded seals hauled out in 70–90% ice coverage (compared with 0–70% and 90–100%) [58]. A larger survey of the central Bering Sea south of St. Lawrence Island from mid-April to mid-June 2007 similarly examined the relationship between bearded seals and four classes of sea ice concentration: 0–25%, 25–50%, 50–75%, and 75–100% [57]. Given that bearded seals were present, their numbers were greatest in the 75–100% class. A still broader survey of the entire U.S. portion of the Bering Sea from early-April to mid-May 2012 showed peak abundance of bearded seals in ice concentrations between about 50% and 75% [59]. Taken together, these studies corroborate our model results indicating that bearded seals choose moderate to high ice concentrations. It is noteworthy, however, that the literature [24, 60] and maps of abundance based on the 2012 survey show bearded seal densities highest towards the northern Bering Sea at the time of maximum sea-ice extent [59]. One possibility is that young bearded seals select habitat differently than adults. None of the analyses based on these aerial surveys accounted for the age of the seal. Yet, as is true for all long-lived species, the majority of the population is composed of older individuals. If there are age-related differences in habitat use those differences could be masked in analyses based on aerial surveys that combine all age classes. In other words, our results, combined with those from aerial surveys, suggest that in the Bering Sea from late fall to early spring, younger bearded seals may tend to select ice edge habitat, while older seals may tend to select areas of higher ice concentration farther from the edge. In the 2007 study, the sea-ice class with the second highest numbers of seals was 0–25% [57]. This range includes our definition of an ice edge (i.e., 10% concentration). If there are spatial differences in the springtime distributions of bearded seals based on age, then it seems likely that most young bearded seals would occupy this sea-ice class (i.e., at the southern edge of the sea-ice). If a bearded seal’s age could be estimated (e.g., based on length) from the images used to survey seals in 2012 [59], a reanalysis of those images could be used to investigate differences in bearded seal distribution based on age.

Presumably older bearded seals select locations with higher ice concentrations farther from an ice edge for some fitness benefit. Perhaps the quality of the benthic foraging habitat [61], the prey assemblages or the protection from killer whales (*Orcinus orca*) [62] are greater in the northern Bering Sea than at the southern edge of the sea-ice. If so, the older animals may simply displace the younger ones from those more beneficial areas. Alternatively, the sea ice habitat itself may be limiting these younger seals from these areas. Although bearded seals tend to occupy sea-ice habitat with natural access to the water, observations indicate that bearded seals are able to make breathing holes in thinner ice [5, 11, 12, 22, 25]. Fay [23] reported that adult bearded seals can use their heads to break holes in ice that is up to 10 cm thick and can maintain those holes in still heavier ice. It is possible, therefore, that a younger bearded seal is incapable of maintaining sufficient breathing holes or water access holes in areas occupied by older seals and so select the ice margins, where the more broken nature of the ice provides reliable spaces for breathing, hauling out, and quick escape back into the water.

There are many cases of young animals behaving differently than their older conspecifics. Another ice-associated seal in Alaska, the ringed seal (*Pusa hispida*), occupied areas with high (near 100%) ice concentrations farther from an ice edge than what was selected by younger animals [63]. The authors hypothesized that adults chose areas with more stable sea-ice on which to build their subnivean lairs for pupping and that younger ringed seals, unconstrained by the need to maintain territories, moved to the Bering Sea ice edge where there are better pelagic feeding opportunities.

Bearded seals do not create subnivean lairs and we were not investigating the pupping season, but previous research has reported differences in the diet of bearded seals based on age [18, 64, 65]. Examinations of stomach contents [18] and tissue chemistries [64] suggest that younger animals are more likely to consume shrimp and less likely to consume clams than older bearded seals. Indeed, benthic surveys of the Bering Sea in mid- to late-summer 2010 suggest a generalized trend of higher densities of shrimp in the southern and eastern Bering Sea than in the north [66]. It seems likely therefore, that prey (type and biomass) would also be important habitat selection factors for bearded seals. Unfortunately, we did not include prey as a covariate in our model because current information on the distributions and abundances of various prey taxa are limited to ice-free periods and locations and so do not appropriately reflect our study area. Until, benthic surveys of the Bering Sea can be conducted in areas with sea ice perhaps sediment type could be used as a proxy for prey type in future models [67, 68].

Although bearded seals are a long-lived species, their population dynamics are likely to be driven by juvenile survival. It has been estimated that only 28% of bearded seals in Alaska survive to age 3 [7]. If persistent, even small reductions in their year-to-year survival could have significant impacts on their overall population abundance. The predicted loss of sea-ice habitat resulting from a warming climate was a primary factor leading to the species listing as “threatened” in U.S. waters, under the ESA [27]. It may therefore, appear fortunate that young bearded seals choose ice edge habitat (that will always be available as long as there is any sea-ice present). As the extent of sea-ice is reduced, however, the location of the ice edge will shift northwards and, given the geography of the Bering Sea, the total area of ice edge habitat will be greatly reduced until the ice edge recedes into the northern Chukchi Sea. These changes in the distribution of sea-ice could require bearded seals to adapt to novel, and possibly suboptimal, conditions and to exploit habitats to which they may not be well suited, potentially compromising their resiliency.

## Conclusion

As expected, shortly after being released in early autumn, most of the bearded seals in our study moved south with the advancing sea-ice through the Bering Strait and into the Bering Sea where they spent the winter and early spring. Our habitat-use model indicated that these young seals selected locations near ice edges. This is in contrast to observations from aerial surveys that showed bearded seals tended to occupy areas farther north and in heavier pack ice at this time. We hypothesize that this discrepancy is the result of age-related behavioral differences between the young animals in our study and the predominantly adult seals in the surveys. Ice concentration was also shown to be a statistically significant variable in our model. All else being equal, areas of higher ice densities are selected for, up to about 80% concentration. The effects of sex or bathymetry were not statistically significant. The close association of young bearded seals to the ice edge in the Bering Sea is important, given the likely effects of climate warming on the extent of sea-ice and subsequent changes in ice edge habitat.

## Supporting information

S1 File Supplementary information about the movement model. (PDF)

## References

[1] Frost KJ, Cameron MF, Simpkins M, Schaeffer C, Whiting A. Diving behavior, habitat use, and movements of bearded seal (*Erignathus barbatus*) pups in Kotzebue Sound and Chukchi Sea Proceedings of the Sixteenth Biennial Conference on the Biology of Marine Mammals; San Diego, CA: Society for Marine Mammalogy; 2005 p. 98–9.

[2] Gjertz I, Kovacs KM, Lydersen C, Wiig Ø. Movements and diving of bearded seal (*Erignathus barbatus*) mothers and pups during lactation and post-weaning. Polar Biology. 2000;23(8):559–66. PubMed PMID: ISI:000088707400007.

[3] Krafft BA, Lydersen C, Kovacs KM, Gjertz I, Haug T. Diving behaviour of lactating bearded seals (*Erignathus barbatus*) in the Svalbard area. Canadian Journal of Zoology. 2000;78(8):1408–18. PubMed PMID: 4779195.

[4] Heptner LVG, Chapskii KK, Arsen'ev VA, Sokolov VT. Bearded seal. *Erignathus barbatus* (Erxleben, 1777) In: Heptner LVG, Naumov NP, Mead J, editors. Mammals of the Soviet Union Volume II, Part 3—Pinnipeds and Toothed Whales, Pinnipedia and Odontoceti. 2, Part 3. Moscow, Russia: Vysshaya Shkola Publishers; 1976 p. 166–217.

[5] Nelson RR, Burns JJ, Frost KJ. The bearded seal (Erignathus barbatus) In: Burns JJ, editor. Marine Mammal Species Accounts, Wildlife Technical Bulletin No 7. 7 Juneau, AK: Alaska Department of Fish and Game; 1984 p. 1–6.

[6] Fedoseev GA. Population structure, current status, and perspective for utilization of the ice-inhabiting forms of pinnipeds in the northern part of the Pacific Ocean In: Yablokov AV, editor. Marine Mammals. Moscow, Russia: Nauka; 1984 p. 130–46.

[7] Burns JJ, Frost KJ. The natural history and ecology of the bearded seal, *Erignathus barbatus* Environmental Assessment of the Alaskan Continental Shelf Final Reports of Principal Investigators Volume 19 December 1983. 19 Juneau, AK: U.S. Department of Commerce, NOAA, and U.S. Department of the Interior; 1983 p. 311–92.

[8] Lydersen C, Kovacs KM. Behaviour and energetics of ice-breeding, North Atlantic phocid seals during the lactation period. Marine Ecology Progress Series. 1999;187:265–81. PubMed PMID: 4682634.

[9] Burns JJ. Arctic marine mammals In: Perrin WF, Wursig BG, Thewissen JGM, editors. Encyclopedia of Marine Mammals. First ed. San Diego, CA: Academic Press; 2002 p. 39–45.

[10] Feltz ET, Fay FH. Thermal requirements in vitro of epidermal cells from seals. Cryobiology. 1966;3(3):261–4. 5970349 10.1016/s0011-2240(66)80020-2

[11] Burns JJ. Bearded seal Erignatus barbatus Erxleben, 1777 In: Ridgway SH, Harrison RJ, editors. Handbook of Marine Mammals Volume 2: Seals. New York, NY: Academic Press; 1981 p. 145–70.

[12] Fedoseev GA. The ecology of the reproduction of seals on the northern part of the Sea of Okhotsk. Izvestiya TINRO. 1965;65:212–6.

[13] Smith TG. Notes on the bearded seal, Erignathus barbatus, in the Canadian Arctic. Quebec, Canada: Department of Fisheries and Oceans, Arctic Biological Station, 1981 0706–6457 Contract No.: 1042.

[14] Kingsley MCS, Stirling I, Calvert W. The distribution and abundance of seals in the Canadian high Arctic, 1980–82. Canadian Journal of Fisheries and Aquatic Sciences. 1985;42(6):1189–210. PubMed PMID: 979375.

[15] Burns JJ, Harbo SJ. An aerial census of spotted seal, *Phoca vitulina largha*, and walruses, *Odobenus rosmarus*, in the ice front of Bering Sea Environmental Assessment of the Alaskan Continental Shelf Quarterly Reports of Principal Investigators April-June 1977 Volume 1. 1 Boulder, CO: U.S Department of Commerce, NOAA and the U.S. Department of Interior, Bureau of Land Management; 1977 p. 58–132.

[16] Finley KJ, Evans CR. Summer diet of the bearded seal (*Erignathus barbatus*) in the Canadian High Arctic. Arctic. 1983;36(1):82–9. PubMed PMID: 514885.

[17] Antonelis GA, Melin SR, Bukhtiyarov YA. Early spring feeding habits of bearded seals (*Erignathus barbatus*) in the Central Bering Sea, 1981. Arctic. 1994;47(1):74–9. PubMed PMID: ISI:A1994NF03600009.

[18] Lowry LF, Frost KJ, Burns JJ. Feeding of bearded seals in the Bering and Chukchi Seas and trophic interaction with Pacific walruses. Arctic. 1980;33(2):330–42.

[19] Kovacs KM. Bearded seal *Erignathus barbatus* In: Perrin WF, Würsig B, Thewissen JGM, editors. Encyclopedia of Marine Mammals. First ed. San Diego, CA: Academic Press; 2002 p. 84–7.

[20] Fedoseev GA. Population biology of ice-associated forms of seals and their role in the northern Pacific ecosystems. Yablokov AV, editor. Moscow, Russia: Center for Russian Environmental Policy, Russian Marine Mammal Council; 2000. 271 p.

[21] Kosygin GM. Feeding of the bearded seal *Erignathus barbatus nauticus* (Pallas) in the Bering Sea during the spring-summer period. Izvestiya TINRO. 1971;75:144–51.

[22] Burns JJ. The Pacific bearded seal. Juneau, AK: Alaska Department of Fish and Game, 1967.

[23] Fay FH. The role of ice in the ecology of marine mammals of the Bering Sea In: Hood DW, Kelley EJ, editors. Oceanography of the Bering Sea. Hakodate, Japan: Institute of Marine Science; 1974 p. 383–99.

[24] Braham HW, Burns J, J., Fedoseev GA, Krogman BD. Distribution and density of ice-associated pinnipeds in the Bering Sea. to be published 1981/1982 as a book Seattle, WA: NOAA, National Marine Fisheries Service, Northwest and Alaska Fisheries Center, National Marine Mammal Laboratory, 1981.

[25] Burns JJ, Frost KJ. The natural history and ecology of the bearded seal, Erignathus barbatus Fairbanks, Alaska: Alaska Department of Fish and Game, 1979 1979. Report No.: Contract No.: Contract #02-5-002-53.

[26] Cameron MF, Bengtson JL, Boveng PL, Jansen JK, Kelly BP, Dahle SP, et al Status review of the bearded seal (Erignathus barbatus) Seattle, WA: U.S. Department of Commerce, 2010.

[27] National Marine Fisheries Service. Endangered and threatened species; threatened status for the Beringia and Okhotsk distinct population segments of the Erignathus barbatus nauticus subspecies of the bearded seal. Federal Register2012. p. 76739–68.

[28] Manly BFJ, McDonald LL, Thomas DL, McDonald TL, Erickson WP. Resource selection by animals: statistical design and analysis for field studies. Nordrecht, Netherlands: Kluwer Academic Publishers; 2002.

[29] R Core Development Team. R: A language and environment for statistical computing. Vienna, Austria: R Foundation for Statistical Computing; 2015.

[30] Whiting A, Griffith D, Jewett S, Clough L, Ambrose W. Combining Inupiaq and scientific knowledge: ecology in northern Kotzebue Sound, Alaska. Fairbanks, AK: Alaska Sea Grant Program, University of Alaska Fairbanks; 2011. 66 p.

[31] Quakenbush L, Citta J, Crawford J. Biology of the bearded seal (Erignathus barbatus) in Alaska, 1961–2009. Fairbanks, AK: Arctic Marine Mammal Program, Alaska Department of Fish and Game, 2011.

[32] Service Argos. Argos User’s Manual. CLS. 2013.

[33] Boyd JD, Brightsmith DJ. Error properties of Argos satellite telemetry locations using least squares and Kalman filtering. PLoS ONE. 2013;8(5):e63051 doi: 10.1371/journal.pone.0063051 23690980 10.1371/journal.pone.0063051 PMC3656847

[34] Freitas C, Lydersen C, Fedak MA, Kovacs KM. A simple new algorithm to filter marine mammal Argos locations. Marine Mammal Science. 2008;24(2):315–25.

[35] Douglas DC, Weinzierl R, C. Davidson S, Kays R, Wikelski M, Bohrer G. Moderating Argos location errors in animal tracking data. Methods Ecol Evol. 2012;3(6):999–1007. doi: 10.1111/j.2041-210X.2012.00245.x

[36] Hosmer DW, Lemeshow S, Sturdivant RX. Applied logistic regression: third edition. New York, NY: John Wiley & Sons; 2013.

[37] Boyce MS. Scale for resource selection functions. Divers Distrib. 2006;12(3):269–76. doi: 10.1111/j.1366-9516.2006.00243.x

[38] Johnson DS, Thomas DL, Hoef JMV, Christ A. A general framework for the analysis of animal resource selection from telemetry data. Biometrics. 2008;64(3):968–76. doi: 10.1111/j.1541-0420.2007.00943.x PubMed PMID: ISI:000258470600034. 18047525 10.1111/j.1541-0420.2007.00943.x

[39] Manly BFJ. Randomization, Bootstrap and Monte Carlo Methods in Biology. London, UK: Chapman Hall; 1997. 399 p.

[40] Blackwell PG. Random diffusion models for animal movement. Ecological Modelling. 1997;100(1–3):87–102. doi: 10.1016/S0304-3800(97)00153-1 PubMed PMID: ISI:000072095000006.

[41] Morales JM, Haydon DT, Frair J, Holsiner KE, Fryxell JM. Extracting more out of relocation data: building movement models as mixtures of random walks. Ecology. 2004;85(9):2436–45. doi: 10.1890/03-0269 PubMed PMID: ISI:000224379600011.

[42] Jonsen ID, Flemming JM, Myers RA. Robust state-space modeling of animal movement data. Ecology. 2005;86(11):2874–80.

[43] Christ A, Ver Hoef JM, Zimmerman DL. An animal movement model incorporating home range and habitat selection Environmental and Ecological Statistics. 2008;15(1):27–38. Epub 19 September 2007 doi: 10.1007/s10651-007-0036-x

[44] Johnson DS, London JM, Lea M-A, Durban J. Continuous-time correlated random walk model for animal telemetry data. Ecology. 2008;89(5):1208–15. 18543615 10.1890/07-1032.1

[45] McClintock B, Johnson D, Hooten M, Ver Hoef J, Morales J. When to be discrete: the importance of time formulation in understanding animal movement. Movement Ecology. 2014;2(1):21 doi: 10.1186/s40462-014-0021-6 25709830 10.1186/s40462-014-0021-6 PMC4337762

[46] Hooten MB, Johnson DS, McClintock BT, Morales JM. Animal movement: statistical models for telemetry data. Boca Raton, FL, USA: CRC Press / Taylor & Francis Group; 2017. 306 p.

[47] Forester JD, Im HK, Rathouz PJ. Accounting for animal movement in estimation of resource selection functions: sampling and data analysis. Ecology. 2009;90(12):3554–65. doi: 10.1890/08-0874.1 PubMed PMID: ISI:000272700800025. 20120822 10.1890/08-0874.1

[48] Potts JR, Bastille-Rousseau G, Murray DL, Schaefer JA, Lewis MA. Predicting local and non-local effects of resources on animal space use using a mechanistic step selection model. Methods Ecol Evol. 2014;5(3):253–62. doi: 10.1111/2041-210X.12150 PubMed PMID: ISI:000332517100006. 25834721 10.1111/2041-210X.12150 PMC4375923

[49] Hooten MB, Johnson DS, Hanks EM, Lowry JH. Agent-based inference for animal movement and selection. J Agric Biol Envir S. 2010;15(4):523–38. doi: 10.1007/s13253-010-0038-2 PubMed PMID: ISI:000284852500007.

[50] Barnard GA. Discussion on Professor Bartlett's paper, 'The spectral analysis of point processes'. Journal of the Royal Statistical Society, Series B. 1963;25:294.

[51] U.S. Department of Commerce, National Oceanic and Atmospheric Administration, Center NGD. 2-minute gridded global relief data (ETOPO2v2) 2006. Available from: http://www.ngdc.noaa.gov/mgg/global/etopo2.html.

[52] R Development Core Team. R: A language and environment for statistical computing. Vienna, Austria: R Foundation for Statistical Computing; 2008.

[53] Cavalieri D, Markus T, Comiso J. AMSR-E/Aqua Daily L3 12.5 km Brightness Temperature, Sea Ice Conentration, & Snow Depth Polar Grids V002. Digital media Boulder, CO: National Snow and Ice Data Center; 2004, updated daily [June 28 2008]. A<D. Available from: http://nsidc.org/data/ae_si12.html.

[54] Fetterer F, Knowles K, Meier W, Savoie M, Windnagel A. Sea Ice Index, Version 2 Boulder, CO USA: NSIDC: National Snow and Ice Data Center; 2016, updated daily. Available from: http://dx.doi.org/10.7265/N5736NV7.

[55] Eisner L, Hillgruber N, Martinson E, Maselko J. Pelagic fish and zooplankton species assemblages in relation to water mass characteristics in the northern Bering and southeast Chukchi seas. Polar Biology. 2013;36(1):87–113. doi: 10.1007/s00300-012-1241-0 PubMed PMID: ISI:000312729700008.

[56] Lele SR, Merrill EH, Keim J, Boyce MS. Selection, use, choice and occupancy: clarifying concepts in resource selection studies. Journal of Animal Ecology. 2013;82(6):1183–91. doi: 10.1111/1365-2656.12141 24499379 10.1111/1365-2656.12141

[57] Ver Hoef JM, Cameron MF, Boveng PL, London JM, Moreland EE. A spatial hierarchical model for abundance of three ice-associated seal species in the eastern Bering Sea. Statistical Methodology. 2014;17:46–66. doi: 10.1016/j.stamet.2013.03.001

[58] Simpkins MA, Hiruki-Raring LM, Sheffield G, Grebmeier JM, Bengtson JL. Habitat selection by ice-associated pinnipeds near St. Lawrence Island, Alaska in March 2001. Polar Biology. 2003;26(9):577–86. doi: 10.1007/s00300-003-0527-7 PubMed PMID: ISI:000184953500002.

[59] Conn PB, Ver Hoef JM, McClintock BT, Moreland EE, London JM, Cameron MF, et al Estimating multispecies abundance using automated detection systems: ice-associated seals in the Bering Sea. Methods Ecol Evol. 2014;5:1280–93. doi: 10.1111/2041-210X.12127

[60] Fiscus CH, Braham HW. Baseline characterization: marine mammals. 1976.

[61] Grebmeier JM, Bluhm BA, Cooper LW, Danielson SL, Arrigo KR, Blanchard AL, et al Ecosystem characteristics and processes facilitating persistent macrobenthic biomass hotspots and associated benthivory in the Pacific Arctic. Progress in Oceanography. 2015;136:92–114. doi: 10.1016/j.pocean.2015.05.006 PubMed PMID: WOS:000358626900007.

[62] Lowry LF, Nelson RR, Frost KJ. Observations of killer whales, *Orcinus orca*, in western Alaska: sightings, strandings, and predation on other marine mammals. Canadian Field-Naturalist. 1987;101(1):6–12. PubMed PMID: ISI:A1987G680800002.

[63] Crawford JA, Frost KJ, Quakenbush LT, Whiting A. Different habitat use strategies by subadult and adult ringed seals (*Phoca hispida*) in the Bering and Chukchi seas. Polar Biology. 2012;35(2):241–55. doi: 10.1007/s00300-011-1067-1 PubMed PMID: ISI:000298858100009.

[64] Young BG, Loseto LL, Ferguson SH. Diet differences among age classes of Arctic seals: evidence from stable isotope and mercury biomarkers. Polar Biology. 2010;33(2):153–62. doi: 10.1007/s00300-009-0693-3 PubMed PMID: ISI:000273594300003.

[65] Crawford JA, Quakenbush LT, Citta JJ. A comparison of ringed and bearded seal diet, condition and productivity between historical (1975–1984) and recent (2003–2012) periods in the Alaskan Bering and Chukchi seas. Progress in Oceanography. 2015;136:133–50. doi: 10.1016/j.pocean.2015.05.011 PubMed PMID: WOS:000358626900009.

[66] Lauth RR. Results of the 2010 eastern and northern Bering Sea continental shelf bottom trawl survey of groundfish and invertebrate fauna U.S. Department of Commerce, 2011.

[67] Cooper LW, Grebmeier JM, Larsen IL, Egorov VG, Theodorakis C, Kelly HP, et al Seasonal variation in sedimentation of organic materials in the St. Lawrence Island polynya region, Bering Sea. Marine Ecology Progress Series. 2002;226:13–26. PubMed PMID: ISI:000174183400002.

[68] Grebmeier J, Cooper L. PacMARS surface sediment parameters, Version 1.0 2014 [August 2009]. Available from: http://pacmars.eol.ucar.edu/.

[69] Cameron M, London J, Frost K, Whiting A, Boveng P. Satellite telemetry dataset (raw): juvenile bearded and spotted seals, 2004–2006, Kotzebue, Alaska. Research Workspace2017.

