# Supplementary material for: Habitat selection and seasonal movements of young bearded seals (Erignathus barbatus) in the Bering Sea
Source: PLoS One. 2018 Feb 28;13(2):e0192743. doi: 10.1371/journal.pone.0192743 (PMC5830299; doi:10.1371/journal.pone.0192743)
Supplement: S1 File — (PDF) [file pone.0192743.s001.pdf]

Supplementary information about the movement model

Successive animal moves are defined by vectors, which have length and direction, and taken together these vectors been termed a correlated random walk (CRW). To build a model of bearded seal movement, we needed to model the distance that a seal moved, and any persistence in bearing (given the last move). As described in the main text, we conditioned on the time intervals actually observed for each seal. We modeled speed, and then used the relationship $S = D/T$, where S is speed, D is distance, and T is time, to obtain distances for any observed time interval. In exploring the data, we noticed that there was a relationship between speed and time, not only in terms of the mean values, but also the variance. Over a long elapsed time, speed will tend toward the average speed, whereas for shorter elapsed times, speed will vary widely due to both short term variability and inherent location error. The raw speeds, in nautical miles per hour, are plotted as a function of the log of the time lag, in hours, in the top panel of Figure S1.1. The top panel of Figure S1.1 reveals the location error effect. At very short time lags, the speed is dominated by location error. At very long time lags, the speed will be dominated by the distance traveled. To model movement, we needed to fit a curve to these data that allowed us to resample speeds for the time lags observed from tagged seals. We could either model the speed/time-lag mean and variance directly, as seen in Figure S1.1, or try some transformation to stabilize the variance. We chose the latter, which we describe next.

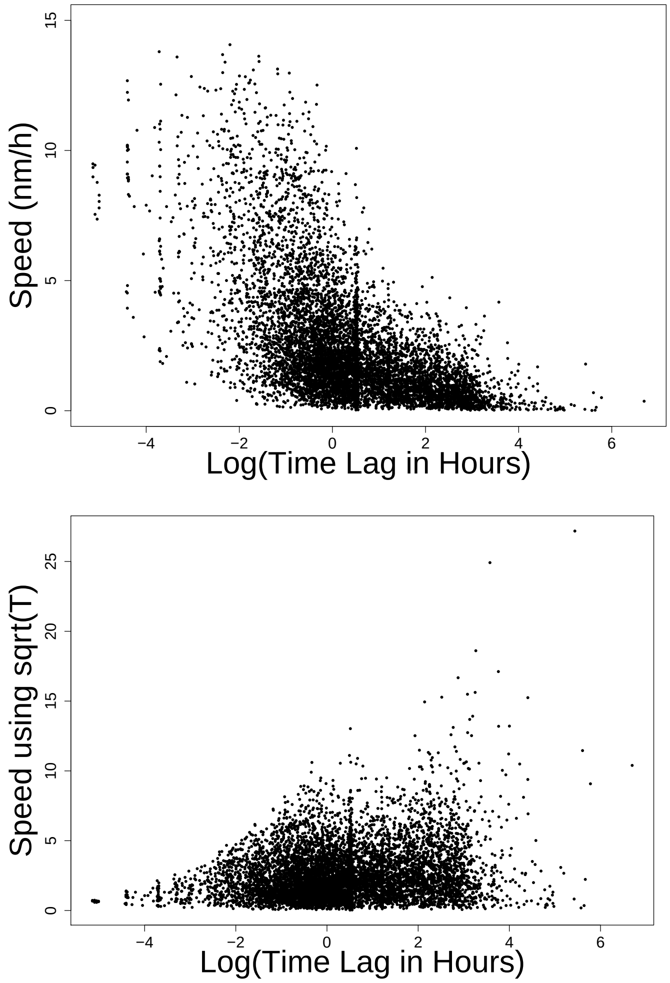

Figure S1.1: The relationship between speed and time, for all animals and all time lags. Top panel, untransformed speed; bottom panel, square-root transformed speed.

Suppose that D is the distance between two locations. Then, assume that distances D_i are independent random variables for very short fixed elapsed times t_i of equal length (all $t_i = t$), and let $\sum_{i=1}^{n} D_i = D$ and $\text{var}(D_i) = t\delta^2$. Then speed S for some elapsed time T is $S = (1/nt)\sum_{i=1}^{n} D_i$, where n and $t_i = t$ are chosen so that $T = \sum_{i=1}^{n} t_i$. Then the variance of S is $nt\delta^2/(n^2t^2) = \delta^2/(nt) = \delta^2/T$. This basic pattern is evident in the top panel in Figure S1.1; the variance decreases as T increases. A first attemp to standardize, following (S1.1),

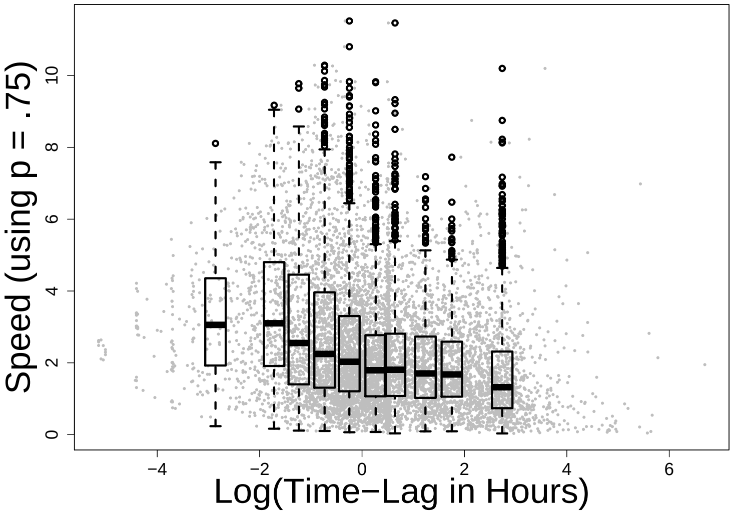

Figure S1.2: The relationship between speed and time for the transformed speeds using $p = 0.75$ in Equation S1.3.

$$\frac{S}{\sqrt{\text{var}(S)}} = \frac{D/T}{\delta/\sqrt{T}} = c\frac{D}{\sqrt{T}}, \qquad \text{(S1.1)}$$

is to simply divide distances by the square-root of their elapsed times (time-lags). This is given in the bottom panel of Figure S1.1. Clearly, the square-root transformation seen in the bottom panel of Figure S1.1 has over-corrected. The reason is that the calculations above assumed the random variables, D_i, were independent at very short time intervals. If we allow them to be perfectly autocorrelated, then calculations similar to those above suggest that standardizing yields

$$\frac{S}{\sqrt{\text{var}(S)}} = \frac{D/T}{\delta} = c\frac{D}{T}. \qquad \text{(S1.2)}$$

Of course, we do not expect distances to be independent, nor perfectly autocorrelated at very short time intervals. The two calculations (S1.1) and (S1.2) suggest that we try a compromise,

$$\frac{S}{\sqrt{\text{var}(S)}} = \frac{D/T}{\delta} = c\frac{D}{T^p}, \qquad \text{(S1.3)}$$

where $0.5 \leq p \leq 1$. We explored various values for p graphically. As shown in Figure S1.2, we found that $p =$

0.75 did reasonably well at stabilizing the variance of speed for all of the time-lags. In Figure S1.2, boxplots are shown over the transformed speed values to help visually assess the mean and variance relationship to elapsed time (time-lag) between successive locations.

After we modeled speed in order to simulate distances for specified times, we needed to model and simulate bearings. We considered two models: relative bearings and absolute bearings. In general, all seals headed south from the Bering Strait, so we chose to model absolute bearings. A histogram of absolute bearings (Figure S1.3) showed a tendency for seals to go south. However, we did expect directional persistence; in other words, we expected that bearings would be temporally auto-correlated. To investigate, we developed transition matrices and tested whether the cell frequencies were independent of the marginal proportions. We created 8 bearing classes centered on 0, 45, 90, 135, 180, 225, 270, and 315 degrees in terms of geographical direction (e.g. 0 is north). For each seal, we then counted the number of transitions from one bearing class to any other bearing class. We then modeled these counts with a generalized linear mixed model, where we included the sex of each seal as a fixed effect, and individual seals as random effects, and assumed a Poisson distribution for the counts. The data were analyzed using the GLIMMIX procedure in the [SAS/STAT] software, Version 9.3.1 of the SAS System for Windows. Copyright © 2008 SAS Institute Inc. SAS and all other SAS Institute Inc. product or service names are registered trademarks or trademarks of SAS Institute Inc., Cary, NC, USA. The results are given in Table S1.1. Note that we only include a main effect for bearing at time i (labeled bearing0 in Table S1.1), but not bearing at time $i+1$ (labeled bearing1 in Table S1.1). This is because, marginally, they are almost exactly identical. If no other effects were signficant in the model, then the fitted values for bearing0 would be very close to those in Figure S1.3. The only reason they would not be identical is that for bearing0 we drop the last record for each seal, in order to use that record for bearing1. Likewise, we only include the interaction of sex with bearing0. If this is signficant, without any higher order interactions, it means that the shape of

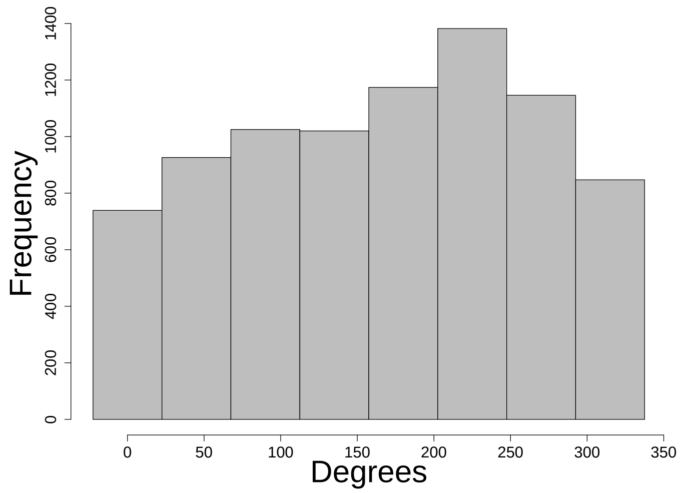

Figure S1.3: Histogram of absolute bearings of all seals movements from observed tracks.

Figure S1.3 is different for each sex. If any further higher order interactions are significant, that implies that there is an interaction between bearing0 and bearing1, so they are not independent. The bearing0*bearing1 test in Table S1.1 shows there is strong evidence for autocorrelation in bearings, and it appears to be different for the sexes because of the significance of the sex*bearing0*bearing1 test.

Table S1.1: Hypothesis tests on the fixed effects in a Poisson regression for the number of bearing transitions.

Type III Tests of Fixed Effects				
Effect	Num DF	Den DF	F Value	Pr > F
sex	1	960	0.27	0.6021
bearing0	7	960	29.45	<.0001
sex*bearing0	7	960	2.63	0.0108
bearing0*bearing1	49	960	4.79	<.0001
sex*bearing0*bearing1	49	960	1.30	0.0187

Table S1.2: Raw proportions of transition bearings for female seals.

		To Bearing							
		0	45	90	135	180	225	270	315
From Bearing	0	0.165	0.150	0.119	0.112	0.104	0.117	0.129	0.104
	45	0.106	0.163	0.142	0.106	0.090	0.155	0.140	0.098
	90	0.084	0.126	0.136	0.103	0.140	0.187	0.138	0.086
	135	0.081	0.078	0.142	0.142	0.185	0.153	0.119	0.100
	180	0.070	0.083	0.095	0.149	0.203	0.184	0.140	0.075
	225	0.069	0.090	0.127	0.098	0.135	0.234	0.167	0.081
	270	0.083	0.130	0.150	0.106	0.117	0.136	0.159	0.119
	315	0.104	0.125	0.144	0.125	0.104	0.174	0.129	0.096

Because there were significant sex effects, we modeled the bearing transition probabilities by using the raw transition proportions by sex. The raw proportional changes from one bearing class to the next for females are given in Table S1.2. Notice that the rows sum to 1. The column sums would provide a scaled version of Figure S1.3, if such a figure were done separately for each sex, and the column sums show that "To Bearing" is generally toward the south. The raw proportional changes from one bearing class to the next for males are given in Table S1.3. In both Table S1.2 and Table S1.3, the temporal autocorrelation in bearing is seen by the tendency for a certain bearing class to have the highest probability of being followed by that same bearing

class; especially for those bearings in the southern direction. This can be seen by the diagonal elements in Table S1.2 and Table S1.3.

Table S1.3: Raw proportions of transition bearings for male seals.

		To Bearing							
		0	45	90	135	180	225	270	315
From Bearing	0	0.120	0.166	0.107	0.123	0.135	0.135	0.104	0.110
	45	0.106	0.114	0.114	0.114	0.151	0.153	0.136	0.111
	90	0.077	0.098	0.143	0.121	0.110	0.185	0.157	0.110
	135	0.088	0.078	0.096	0.155	0.155	0.145	0.151	0.131
	180	0.098	0.088	0.079	0.144	0.190	0.193	0.099	0.109
	225	0.067	0.098	0.102	0.137	0.174	0.202	0.132	0.088
	270	0.087	0.137	0.173	0.131	0.103	0.131	0.131	0.109
	315	0.074	0.117	0.117	0.121	0.133	0.126	0.164	0.148

Next, we investigated speed as a function of bearing. Recall from (S1.3) that the transformation of time that we used to develop speed was $S = D/T^{0.75}$. Initally, we fit a model of S to bearing, but the residuals were highly skewed. We determined that the fifth root of speed gave reasonable residuals. The fitted model is given in Table S1.4, where x is bearing in degrees from 0 to 360, and $x2$ is bearing-squared. The fitted model is

Table S1.4: Fitted model for speed relationship to bearing.

	Estimate	Std. Error	t value	Pr(>|t|)
(Intercept)	1.0974082	0.00628801	174.524	0.00000
x	0.0005714	0.00007860	7.269	0.00000
x2	-0.0000016	0.00000021	-7.244	0.00000

displayed graphically in Figure S1.4. In Figure S1.4A, the solid circles are average values in bearing classes incremented by 10 degrees, and the fitted line is also given. For those same 10 degree bearing classes, boxplots of residuals are presented are Figure S1.4B, which shows a good fit and justifies the fifth-root transformation. The residual standard error of the model was 0.1766.

We took this to be our final model that established relationships between speed, time, distance, and bearing. Transitional bearings are given in Table S1.2 and Table S1.3, and simulating from them is straightforward because each row can be interpreted as a probability. Then, given a bearing, we can obtain speed (or distance)

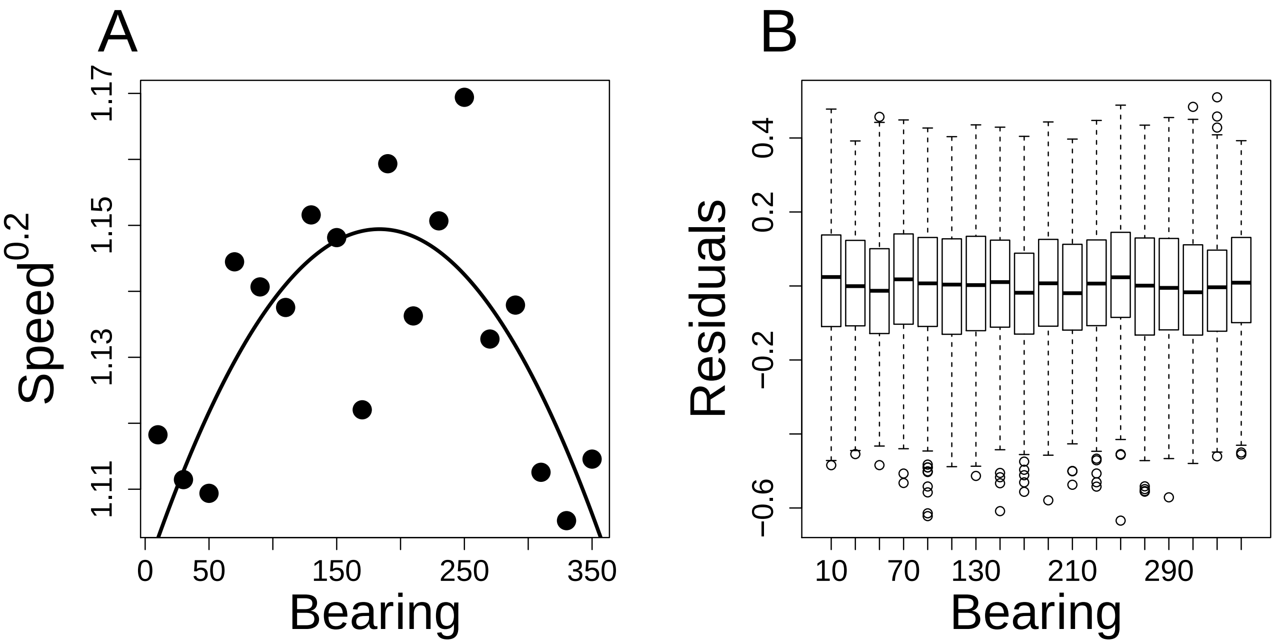

Figure S1.4: Relationship between speed and bearing.

for a given time-lag through the fitted model,

$$S^* = \left(\frac{D}{T^{0.75}}\right)^{0.2} = 1.097 + 0.005714(\text{bearing}) - 0.000001557(\text{bearing})^2, \tag{S1.4}$$

where $S = (S^*)^5$. We can simulate randomly from (S1.4) by using the estimated standard error of 0.1766.

The simulation of bearded seal locations under the null model of a CRW as described above, for each observed seal track, consisted of the following steps:

- Step 1. The simulated track, matched to an observed seal, started with the observed seal's first location.
- Step 2. If it was that individual's first location, the initial bearing was 180 degrees. Otherwise, a random bearing was drawn from the transition probabilities in Table S1.3 and Table S1.2, depending on the individual's sex. After drawing the bearing class, a uniform distribution was used within bearing class to randomly draw an exact bearing.
- Step 3. Given the bearing in Step 2, a random speed was drawn from $\mathrm{N}(S^*, 0.1766^2)$, where S^* was developed in (S1.4), and $\mathrm{N}(\mu, \sigma^2)$ is a normal distribution with mean μ and variance σ^2.

- Step 4. The randomly drawn speed was converted to distance by using $D = (S^*)^5 T^{0.75}$, where T is matched to the time interval between locations for the observed animal.

- Step 5. Move to the new location using the randomly drawn bearing and distance, and go to Step 2. If the new location is on land, or the move crossed land, reject the move and go back to Step 2. If the new location is the last one (matched location by location to the observed animal), stop for that animal and begin a new animal at Step 1.

Notice that each simulated CRW, under the null model of no habitat influences, exactly matches each observed seal in terms of time intervals between movements and total number of locations. Also note that Table S1.3 and Table S1.2 were developed for seals that tended southward (Figure S1.3), and hence simulated tracks also tended southward. Simulated tracks tended towards the Bering Sea because: 1) we started them with a 180 degree bearing, 2) the southward tendency of the transition matrices, and 3) the land mass constriction between the Bering and Chukchi seas, along with the rejection of movements on or across land, required many consecutive, relatively improbable steps to pass into the Chukchi Sea.
